# Inhibitory Activity of a Scorpion Defensin BmKDfsin3 against Hepatitis C Virus

**DOI:** 10.3390/antibiotics9010033

**Published:** 2020-01-17

**Authors:** Yuting Cheng, Fang Sun, Songryong Li, Minjun Gao, Luyao Wang, Moustafa Sarhan, Mohamed A. Abdel-Rahman, Wenxin Li, Hang Fai Kwok, Yingliang Wu, Zhijian Cao

**Affiliations:** 1 State Key Laboratory of Virology, Modern Virology Research Center, College of Life Sciences, Wuhan University, Wuhan 430072, China; yuting920428@whu.edu.cn (Y.C.); 2016202040055@whu.edu.cn (F.S.); 888lichenglong@sina.com (S.L.); gmj1995820@163.com (M.G.); 2018202040036@whu.edu.cn (L.W.); wxli@whu.edu.cn (W.L.); ylwu@whu.edu.cn (Y.W.); 2 Zoology Department, Al-Azhar University, Assuit 71524, Egypt; moustafasarhan@hotmail.com; 3 Zoology Department, Faculty of Science, Suez Canal University, Ismailia 41522, Egypt; mohamed_hassanain@science.suez.edu.eg; 4 Institute of Translational Medicine, Faculty of Health Sciences, University of Macau, Avenida de Universidade, Taipa, Macau 999078, China; hfkwok@um.edu.mo; 5 Hubei Province Engineering and Technology Research, Center for Fluorinated Pharmaceuticals, Wuhan University, Wuhan 430072, China

**Keywords:** scorpion defensin, Hepatitis C virus (HCV), p38

## Abstract

Hepatitis C virus (HCV) infection is a major worldwide health problem which can cause chronic hepatitis, liver fibrosis and hepatocellular carcinoma (HCC). There is still no vaccine to prevent HCV infection. Currently, the clinical treatment of HCV infection mainly relies on the use of direct-acting antivirals (DAAs) which are expensive and have side effects. Here, BmKDfsin3, a scorpion defensin from the venom of *Mesobuthus martensii* Karsch, is found to dose-dependently inhibit HCV infection at noncytotoxic concentrations and affect viral attachment and post-entry in HCV life cycle. Further experimental results show that BmKDfsin3 not only suppresses p38 mitogen-activated protein kinase (MAPK) activation of HCV-infected Huh7.5.1 cells, but also inhibits p38 activation of Huh7.5.1 cells stimulated by tumor necrosis factor-α (TNF-α), interleukin-1β (IL-1β) or lipopolysaccharide (LPS). BmKDfsin3 is also revealed to enter into cells. Using an upstream MyD88 dimerization inhibitor ST2345 or kinase IRAK-1/4 inhibitor I, the inhibition of p38 activation represses HCV replication in vitro. Taken together, a scorpion defensin BmKDfsin3 inhibits HCV replication, related to regulated p38 MAPK activation.

## 1. Introduction

Defensin is a class of cationic peptides rich in disulfide bonds and widely distributed in fungi, plants and animals, and is also an important part of the defense system [1]. Scorpion defensins have a variety of biological activities, including antiviral activity [2,3], antibacterial activity [4,5], immunomodulatory function [6,7], antitumor action [8,9], and ion channel modulation [10,11]. A scorpion defensin BmKDfsin4 derived from the venom of the scorpion *Mesobuthus martensii* Karsch was reported to inhibit hepatitis B virus (HBV) replication by our group [12]. Although such report demonstrated that the scorpion defensin can repress viral production, the specific mechanism of this effect during viral infection is not well understood.

Hepatitis C virus (HCV) infection can cause chronic diseases such as chronic hepatitis, liver cirrhosis, liver fibrosis, and hepatocellular carcinoma (HCC), which seriously threatens human health [13]. The HCV genome is about 9.6 kb in length and translates into a polyprotein precursor of approximate 3000 amino acid residues. This polyprotein precursor is further processed to yield 3 structural proteins (core, E1 and E2) and 7 non-structural proteins (p7, NS2, NS3, NS4A, NS4B, NS5A, and NS5B). The HCV core protein is the first protein to be cleaved, which forms the viral nucleocapsid and encloses the viral ribonucleicacid (RNA) [14]. Due to the limitation of the HCV culture system and the adaptive mutations of the virus, there is currently no vaccine that can prevent HCV infection. The treatment of patients with HCV infection is mainly based on direct-acting antivirals (DAAs). The DAAs currently used in clinical practice include three main categories: NS3/4A protease inhibitors, NS5A inhibitors, and NS5B polymerase inhibitors. Commonly used DAAs are sofosbuvir, daclatasvir, ledipasvir, and velpatasvir etc., but they have some side effects. Sofosbuvir, for example, can cause insomnia, mild headache, and nausea [15]. Additionally, the DAAs have the disadvantages of gene selectivity, the risk of sustained immune response, low accessibility and resistance to mutated virus strains, a long treatment cycle and expensive cost [16]. Therefore, it is extremely important to find anti-HCV targets or new anti-HCV drugs.

Previous studies showed that infections with many viruses such as HCV [17], chikungunya virus (CHIKV) [18], porcine epidemic diarrhea virus (PEDV) [19], herpes simplex virus (HSV) [20], enterovirus 71 (EV71) [21], human immunodeficiency virus (HIV) [22], and dengue virus (DENV) [23], can activate p38 mitogen-activated protein kinase (MAPK). Furthermore, the p38 MAPK inhibitor can inhibit the replication of many viruses, like HSV [24,25], EV71 [21], and CHIKV [18]. Additionally, an α-type scorpion toxin BmK NT1 can induce p38 phosphorylation [26] and a scorpion venom heat-resistant peptide (SVHRP) from *M. martensii* Karsch suppresses the activation of p38 [27], suggesting that scorpion venom peptides can regulate p38 activity by different pathways. Therefore, we ask whether the scorpion defensin BmKDfsin3 affects viral replication and regulates virus-induced p38 activation.

BmKDfsin3, a scorpion defensin derived from *M. martensii* Karsch contains 38 amino acid residues, which includes six cysteine residues forming three pairs of disulfide bonds. During this study, we found that BmKDfsin3 can inhibit HCV replication and affect the attachment and post-entry stages of the viral infection cycle at noncytotoxic concentrations. Then, we observed that p38 activation is suppressed by BmKDfsin3 during HCV infection. Additionally, BmKDfsin3 is revealed to enter into the cells. Expectedly, inhibiting the p38 MAPK signal pathway by using the MyD88 dimerization inhibitor and IRAK inhibitor also can suppress HCV replication. Briefly, BmKDfsin3 inhibits HCV replication, which is related to the classical p38 MAPK signal pathway.

## 2. Results

### 2.1. BmKDfsin3 Inhibits HCV Replication In Vitro at Noncytotoxic Concentrations

**Figure 1:**
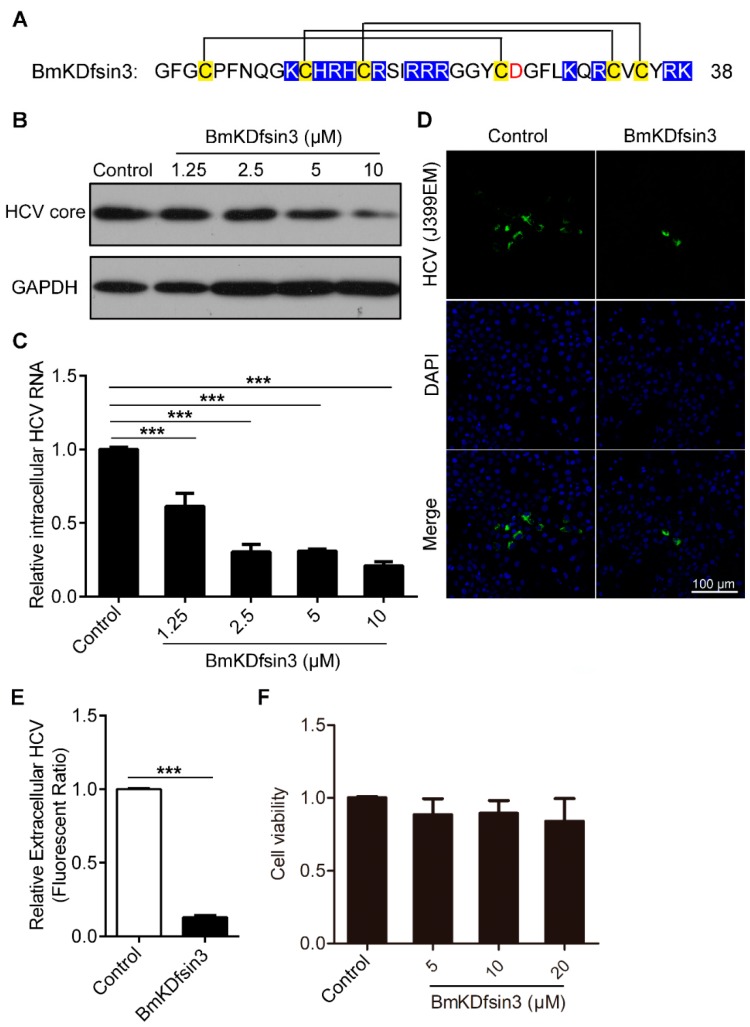
BmKDfsin3 inhibits HCV replication in vitro under noncytotoxic concentrations. (**A**) Amino acid sequence of BmKDfsin3. There are six cysteine residues forming three pairs of disulfide bonds. Cysteine residues are labeled with yellow, basic residues with blue, and acidic residues with red. (**B**,**C**) Concentration-dependent inhibition of BmKDfsin3 on HCV infection in Huh7.5.1 cells. Huh7.5.1 cells were preincubated with the BmKDfsin3 at different concentrations for 1 h and then infected with HCV J399EM at an multiplicity of infection (MOI) of 0.1. After 72 h, cells were collected, and intracellular HCV core protein levels were analyzed by western blotting (**B**) and intracellular HCV RNA was analyzed by qPCR (**C**). The J399EM was derived from the JFH-1 virus (HCV genotype 2a strain) by insertion of eGFP into the HCV NS5A region. (**D**) Inhibition of BmKDfsin3 on extracellular virus particles of Huh7.5.1 cells. Huh 7.5.1 cells treated with or without BmKDfsin3 (5 μM) were infected by J399EM for 72 h, and then supernatant was collected and used to incubate naïve Huh7.5.1 cells for 72 h. Virus in Huh 7.5.1 cells were observed by immunofluorescence microscope. HCV, green. 4’,6-diamidino-2-phenylindole (DAPI), blue. Scale bar, 100 μm. (**E**) The statistics of the fluorescence ratio of extracellular virus particles as described in (**D**). (**F**) Cytotoxicity of BmKDfsin3 to Huh7.5.1 cells by the MTT assay. BmKDfsin3 was dissolved in the medium and the medium without BmKDfsin3 was used as a negative control in all experiments. The internal control of subfigure (**C**) was glyceraldehyde-phosphate dehydrogenase (GAPDH). ***, *p* < 0.001. Data represented the mean ± standard deviation (SD) of at least three independent experiments.

BmKDfsin3 is a scorpion defensin characterized in the *M. martensii* genome [28]. The linear formation of BmKDfsin3 was chemically synthesized. The linear BmKDfsin3 was then folded by an air oxidation method. There are six cysteine residues forming three pairs of disulfide bonds, C1–C4, C2–C5 and C3–C6, respectively (Figure 1A). To determine the antiviral activity of BmKDfsin3 against HCV infection, we analyzed the intracellular core protein and RNA of HCV and the extracellular virus in Huh7.5.1 cells treated with or without BmKDfsin3. The results of western blotting showed that BmKDfsin3 reduced the expression level of HCV core protein, and its inhibition rates were 18%, 49%, 58%, and 86% at the concentrations of 1.25 μM, 2.5 μM, 5 μM, and 10 μM, respectively (Figure 1B). The 50% inhibitory concentration (IC_50_) was 3.35 ± 1.1 μM calculated by GraphPad Prism 5 (GraphPad Software, Inc., San Diego, CA, USA). Intracellular HCV RNA also was suppressed by BmKDfsin3 in a concentration-dependent manner as shown by quantitative PCR (qPCR) (Figure 1C). Additionally, we found that the supernatant of Huh7.5.1 cells treated with BmKDfsin3 had less green fluorescence than the supernatant of cells treated without BmKDfsin3 (Figure 1D,E), suggesting that the supernatant of Huh7.5.1 cells treated with BmKDfsin3 has less HCV J399EM particles than that treated without BmKDfsin3. The cytotoxic effect of BmKDfsin3 was analyzed by an 3-[4,5-dimethylthiazol-2-yl]-2,5 diphenyl tetrazolium bromide (MTT) cytotoxicity assay in Huh7.5.1 cells, and its CC_50_ to Huh7.5.1 cells was 60.63 ± 1.36 μM. The results show that BmKDfsin3 was substantially noncytotoxic to Huh7.5.1 cells at the used concentrations during antiviral experiments (Figure 1F). Taken together, BmKDfsin3 inhibits HCV replication at noncytotoxic concentrations in vitro.

### 2.2. BmKDfsin3 Affects the Viral Attachment and Post-Entry Stages in HCV Infection Cycle

**Figure 2:**
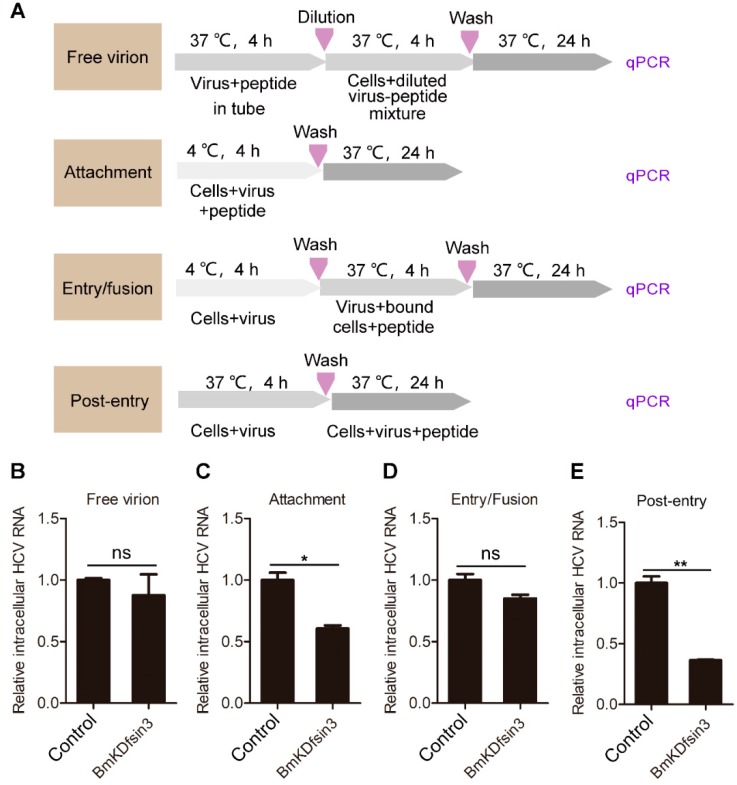
BmKDfsin3 affects the attachment and post-entry stages during the HCV infection cycle. (**A**) Schematic diagram for studying the action stage of BmKDfsin3 on HCV. (B–E) The effects of BmKDfin3 on free virion (**B**), attachment (**C**), entry/fusion (**D**), and post-entry (**E**) stages of HCV in Huh7.5.1 cells. Huh7.5.1 cells were infected with J399EM at an MOI of 0.1 and treated with BmKDfsin3 as described in (**A**). All experiments were detected by qPCR. BmKDfsin3 was dissolved in the medium and the medium without BmKDfsin3 was used as a negative control. The internal controls of subfigures (**B**–**E**) were GAPDH. ns, no significance. *, *p* < 0.05. **, *p* < 0.01. Data represented the mean ± SD of at least three independent experiments.

We have proven that the scorpion defensin BmKDfsin3 concentration-dependently inhibits HCV replication under noncytotoxic concentrations in Huh7.5.1 cells. To determine the action stage of the peptide BmKDfsin3 during the HCV life cycle, we conducted an experiment with different modes of peptide treatment, shown in the schematic diagram of Figure 2A. The experimental results indicated that BmKDfin3 had almost no effect on the free virion (Figure 2B) and viral entry/fusion (Figure 2D) stages but had 42% inhibition on the viral attachment stage (Figure 2C). Importantly, BmKDfsin3 added during the post-entry stage had significant restriction (64%) on HCV replication in Huh7.5.1 cells (Figure 2E). These data suggest that BmKDfsin3 suppresses HCV replication by acting on the viral attachment and post-entry phases, and the latter is more effective than the former.

### 2.3. BmKDfsin3 Inhibits p38 Activation

**Figure 3:**
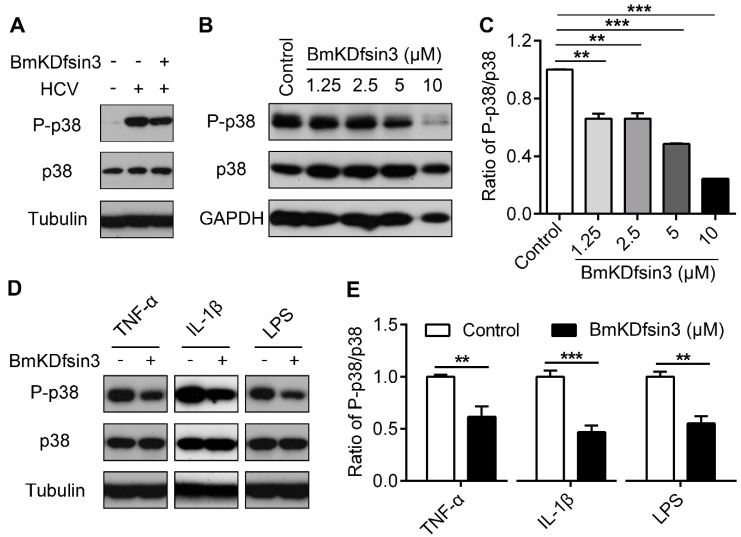

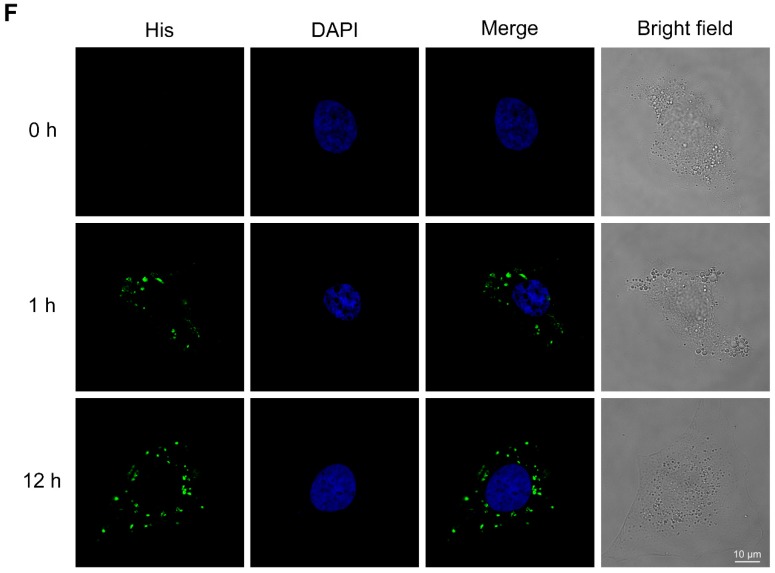
BmKDfsin3 inhibits p38 activation. (**A**–**C**) BmKDfsin3 suppresses p38 phosphorylation in HCV-infected Huh7.5.1 cells. Huh7.5.1 cells were infected with J399EM at an MOI of 1 for 2 h, and then treated with BmKDfsin3 (5 μM) following the detection of intracellular total p38, phosphorylated p38 and HCV core protein levels by western blotting (**A**). Huh7.5.1 cells were treated as (**A**) and then with the BmKDfsin3 at different concentrations. The expression levels of total p38 and phosphorylated p38 were determined by western blotting (**B**). Grayscale of Figure B was analyzed with Image J software (**C**). BmKDfsin3 was dissolved in the medium and the medium without BmKDfsin3 was used as a negative control. (**D**,**E**) Effect of BmKDfsin3 on p38 activation in Huh7.5.1 cells stimulated by TNF-α, IL-1β or LPS. Huh7.5.1 cells were incubated with TNF-α (100 ng/mL), IL-1β (1 ng/mL), and LPS (1 μg/mL) (**E**) for 2 h, and then treated by BmKDfsin3 (5 μM). Phosphorylation level of p38 was detected by western blotting. Grayscale of Figure D was analyzed with Image J software (**E**). TNF-α, IL-1β, and LPS were dissolved in phosphate buffered saline (PBS) and PBS was used as a negative control. **, *p* < 0.01. ***, *p* < 0.001. Data represented the mean ± SD of at least three independent experiments. (**F**) The entry of His-BmKDfsin3 to Huh7.5.1 cells. Cells were treated with His-BmKDfsin3 (5 μM) for 0 h, 1 h and 12 h, respectively, and then stained with anti-His antibody and DAPI. Cells were observed using a confocal microscopy. His-BmKDfsin3, green. DAPI, blue. Scale bar, 10 μm. Cells were treated with His-BmKDfsin3 (5 μM) for 0 h as a control. The subfigure (**F**) was representative of at least ten independent pictures.

Many previous studies have reported that the infection of host cells with some viruses including HCV [17] can activate p38. Here, we found that HCV infection increased p38 phosphorylation in Huh7.5.1 cells. Interestingly, the activated p38 was significantly inhibited by the treatment with BmKDfsin3 (Figure 3A). To further confirm this effect, we added different concentrations of BmKDfsin3 to HCV-infected Huh7.5.1 cells and then detected changes of the phosphorylated p38 using western blotting. The results showed that p38 phosphorylation was inhibited during HCV infection in a concentration-dependent manner after the addition of BmKDfsin3 (Figure 3B,C). However, considering that HCV infection can activate p38, it is still unknown whether the inhibitory effect of BmKDfsin3 on p38 activation results from its anti-HCV activity or its effect on the p38 cascade. To analyze the effect of BmKDfsin3 on the p38 cascade, we used three p38 activation stimulation factors tumor necrosis factor-α (TNF-α), interleukin-1β (IL-1β) and lipopolysaccharide (LPS) [29] to incubate Huh7.5.1 cells for 2 h, and then treated the cells with BmKDfsin3. We analyzed the phosphorylation of p38 by western blotting. Results showed that BmKDfsin3 significantly inhibited the phosphorylation of p38 induced by TNF-α, IL-1β and LPS (Figure 3D,E). Additionally, we also chemically synthesized an His tag-fused BmKDfsin3 (His-BmKDfsin3) and incubated it with Huh7.5.1 cells for 0 h, 1 h and 12 h, respectively. Then, the entry of His-BmKDfsin3 in Huh7.5.1 cells was analyzed using a confocal microscope. The results showed that His-BmKDfsin3 could enter Huh7.5.1 cells (Figure 3F). Taken together, these data suggest that BmKDfsin3 inhibits p38 activation during HCV infection, which is possibly related to regulation of the p38 MAPK signal pathway.

### 2.4. Inhibition of p38 Activation Suppresses HCV Replication In Vitro

**Figure 4:**
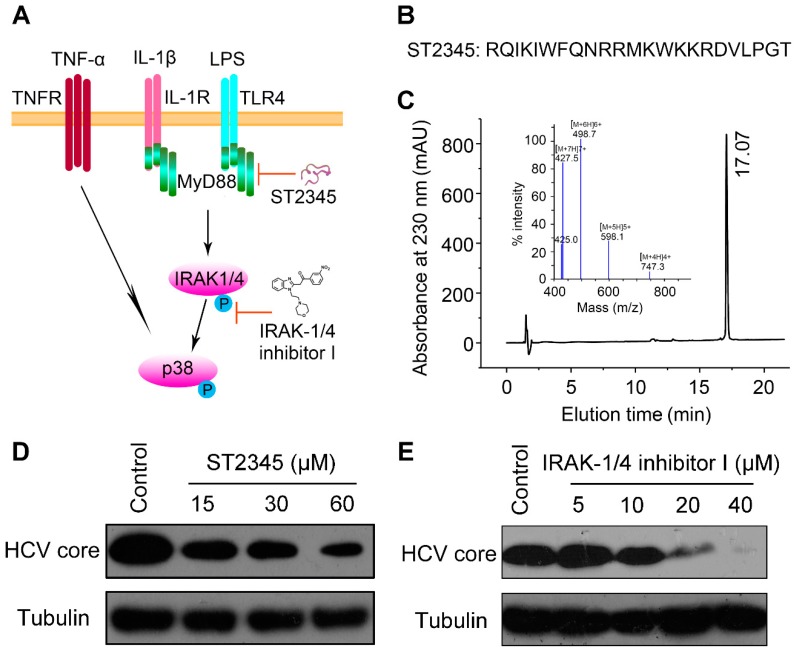
Inhibition of p38 activation suppresses HCV replication in vitro. (**A**) Schematic diagram of the p38 MAPK signal pathway and its inhibiting by ST2345 (MyD88 inhibitor) and IRAK-1/4 inhibitor I (IRAK1 and IRAK4 inhibitor). (**B**) Amino acid sequence of ST2345. (**C**) Mass spectrometry and HPLC analysis of ST2345 (1 μg/μL). (**D**,**E**) The inhibition of ST2345 and IRAK-1/4 inhibitor I on HCV infection. Huh7.5.1 cells were infected with J399EM at an MOI of 0.1, and then treated with different concentrations of ST2345 (**D**) and IRAK-1/4 inhibitor I (**E**) for 72 h, respectively. The expression level of the HCV core protein was determined by western blotting. The ST2345 peptide was dissolved in medium and the medium without ST2345 was used as a negative control. The IRAK-1/4 inhibitor I was dissolved in dimethyl sulfoxide (DMSO) and the DMSO without IRAK-1/4 inhibitor I was used as a negative control.

Next, we ask whether the p38 signaling pathway played an important role in HCV replication. MyD88 and IRAK1/4 are two important upstream cascade proteins of the p38 signaling pathway, as shown in Figure 4A. ST2345 is a polypeptide with 23 amino acid residues (Figure 4B) that interferes with MyD88 toll/IL-1R (TIR) domain dimerization [30]. ST2345 peptide was chemically synthesized by GL Biochem Ltd. (GL Biochem (Shanghai) Ltd. Limited Liability Company, Shanghai, China). High-performance liquid chromatography (HPLC) analysis indicated that the elution time of the peptide ST2345 was in 17.07 min at 230 nm and the purity of ST2345 was more than 95% (Figure 4C). Mass spectrometry analysis indicated that the mass of the synthetic ST2345 peptide matches its theoretical molecular weight (2985.60 Da) (Figure 4C). IRAK-1/4 inhibitor I is a small molecule that suppresses the phosphorylations of IRAK1 and IRAK4 with IC_50_ of 0.3 μM and 0.2 μM, respectively [31]. Both ST2345 and IRAK-1/4 inhibitor I can inhibit the activation of p38 by regulating the p38 signaling pathway. Therefore, they were used to investigate anti-HCV activity, respectively. The experimental results showed that the peptide ST2345 significantly inhibited HCV replication in a dose-dependent manner (Figure 4D). Similarly, IRAK-1/4 inhibitor I was observed to have a concentration-dependent antiviral activity against HCV infection in Huh7.5.1 cells (Figure 4E). These results indicate that inhibition of p38 activation suppresses HCV replication and the p38 signaling pathway may play an important role in viral infection.

## 3. Discussion

Many studies have previously reported that defensins have antibacterial, antiviral and ion channel-modulation activities [32,33,34]. Recently, there are many discoveries regarding antiviral defensin polypeptides from scorpions, which may be new candidates for antiviral drug molecules. Human α- and β-defensins can suppress HCV replication [35]. Additionally, human α-defensin-1 inhibited protein kinase C (PKC) activation and repressed influenza virus replication [36]. Recent published data also suggest that α-defensin-1 may not only act directly on the virus in the absence of serum, but on the cell in the presence of serum as well [37]. Previous studies in our laboratory also found that the scorpion defensin BmKDfsin4 inhibited HBV replication [12] and blocked potassium channels [10]. Additionally, Zeng et al. designed a histidine-rich Eval418 derivative that could significantly inhibit herpes simplex virus 1 (HSV-1) replication [38]. During our study, the scorpion defensin BmKDfsin3 was found to be capable of inhibiting HCV replication in a concentration-dependent manner under non-cytotoxicity. BmKDfsin3 is a new antiviral peptide and our study provides a new molecule for anti-HCV.

HCV infection can activate the p38 MAPK [17]. p38 MAPK, a member of the mitogen-activated protein (MAP) kinase family, plays crucial roles in many biological processes in response to extracellular stimuli that mediate a variety of cellular behaviors. p38 activated by many extracellular signals executes important physiological and pathological functions in inflammation, cell proliferation, apoptosis, differentiation, aging and tumorigenesis and, especially, cellular stress and infection [39,40]. Classical p38 activation follows the activation of the MAPK signaling molecules MAP kinase kinase kinase (MAP3K), MAP kinase kinase (MAPKK), and MAP kinase (MAPK) cascades. The MKKs of p38 in this pathway are mainly MKK3 and MKK6 which activate the phosphorylations of Thr180 and Tyr182 in p38, respectively [41]. Previously, respiratory syncytial virus (RSV) was reported to induce Toll-like receptor 4 (TLR4)-mediated activation of p38 in the early stages of infection, which was important for viral infection [42]. p38 specific inhibitor can suppress RSV and influenza A virus replication, and the phosphorylated p38 induced by virus can also be inhibited [43]. Here, BmKDfsin3 was found to inhibit HCV replication. Moreover, BmKDfsin3 was revealed to repress p38 activation. Concurrently, HCV replication can also be suppressed when the p38 MAPK signal pathway is inhibited by the treatment of the MyD88 inhibitor or IRAK inhibitor. All these suggest that a scorpion defensin BmKDfsin3 inhibits HCV replication, related to regulated p38 MAPK activation.

## 4. Materials and Methods

### 4.1. Reagents and Antibodies

Scorpion defensin BmKDfsin3, His-BmKDfsin3 and ST2345 peptide were synthesized by GL Biochem (Shanghai, China). TNF-α (96-300-01A-10) and IRAK-1/4 inhibitor I (HY-13329) were purchased from the PeproTech (Rocky Hill, CT, USA) and MedChemExpress (Monmouth Junction, NJ, USA), respectively. IL-1β (SRP6169) and LPS (L2630) were purchased from Sigma-Aldrich (St. Louis, MO, USA). Antibodies against P-p38 MAPK (4511), and p38 MAPK (9212) were from the Cell Signaling Technology (Beverly, MA, USA). Anti-HepC cAg (C7-50) (sc-57800) antibody was purchased from Santa Cruz Biotechnology (Delaware Ave Santa Cruz, CA, USA). Antibodies against GAPDH (60004-1-lg), Tubulin (11224-1-AP), and Flag (20543-1-AP) were from ProteinTech Group (Wuhan, China). Alexa Fluor 488 Affinipure donkey anti-mouse IgG (H+L) (34106ES60) was purchased from Yeasen (Shanghai, China).

### 4.2. Cells and Viruses

Huh7.5.1 and HEK293T cells were cultured in Dulbecco’s modified Eagle’s medium (Gibco-Invitrogen) supplemented with 10% fatal bovine serum (FBS) (Gibco-Invitrogen, Foster, CA, USA) and 1% penicillin/streptomycin. Cells were cultured with humidified 5% CO_2_ at 37 °C. The J399EM was derived from the JFH-1 virus strain (HCV genotype 2a) by insertion of eGFP into the HCV NS5A region and was kindly provided by Professor Xinwen Chen (Wuhan Institute of Virology, CAS) [44].

### 4.3. MTT Assay

To analyze the cytotoxic effect of BmKDfsin3 on Huh7.5.1 cells, we used the MTT cytotoxicity assay, as in the previous article published in our laboratory [38]. Briefly, Huh7.5.1 cells were planted in 96-well plates (10^4^ cells per well) and cultured at 37 °C for 24 h. The BmKDfsin3 was diluted with fresh medium in different concentrations and then replaced the cell culture medium, and the cells were cultured at 37 °C for 48 h. Then, the medium was removed, and the cells were incubated with the 0.25 μg/μL MTT solution in 200 μL of medium per each well at 37 °C for 4 h. The medium was then replaced with 100 μL DMSO and shaken gently for 10 min at room temperature. The absorbance was then measured at a wavelength of 570 nm using a microplate reader (BioTek, Winooski, VT, USA).

### 4.4. Confocal Microscopy

To detect infectious HCV virions in the supernatant of HCV-infected cells treated with BmKDfsin3 or not, the collected supernatants were used to infect naïve Huh7.5.1 cells. The cells were fixed with precooled 4% paraformaldehyde, and the nuclei were stained with DAPI (ANT10072). The green fluorescence (HCV J399EM) was observed under a confocal microscope.

To test whether BmKDfsin3 can enter into cells, we added His-tagged BmKDfsin3 (5 μM) to Huh7.5.1 cells and incubated them in confocal dishes for 0 h, 1 h and 12 h, respectively. These cells were washed three times with cold PBS and fixed with precooled 4% paraformaldehyde and permeated with 0.2% Triton X-100. The cells were washed three times with PBS and then blocked with 8% body surface area (BSA) for 1 h at room temperature. Then, cells were incubated with 1% BSA diluted primary antibody and shaken overnight at 4 °C. Subsequently, the cells were incubated with the secondary antibody Alexa Fluor 488 for 1 h at room temperature in the dark, and then the nuclei were stained with DAPI and observed under a confocal microscope.

### 4.5. qPCR

The method of qPCR for detecting intracellular viral RNA was consistent with that of our laboratory [45]. Briefly, the detected cells were lysed using TRIzol reagent (Takara) to release the intracellular total RNA, followed by precipitation with isopropanol. The first-strand cDNA was reversed-transcribed by the RevertAid first strand cDNA synthesis kit (ThermoFisher Scientific). The cDNA was quantitated by qPCR with primers using the Bestar^®^ SYBR Green qPCR master mix reagent (DBI^®^ Bioscience). HCV primers were 5′-TCTGCGGAACCGGTGAGTA-3′ (sense) and 5′-TCAGGCAGTACCACAAGGC-3′ (antisense).

### 4.6. Western Blotting

Cells were collected and lysed 30 min on ice by lysis buffer (1% TritonX-100, 10% glycerol, 50 mM HEPES, pH 7.2, 150 mM NaCl). The cell lysate was centrifuged at 12,000 rpm for 15 min, and then the supernatant was collected and assayed with a bicinchoninic acid (BCA) protein quantification kit (ThermoFisher Scientific, Cleveland, OH, USA). Samples were separated by 10% sodium dodecyl sulfate-polyacrylamide gel electrophoresis (SDS-PAGE) and transferred to nitrocellulose (NC) membranes. The non-specific protein on the NC membrane was blocked with 5% skim milk powder and incubated on a shaker for 2 h at room temperature. The primary antibody was incubated overnight at 4 °C, and the secondary antibody was incubated for 2 h at room temperature. The results were analyzed by a chemiluminescence kit with FuJi medical X-ray film.

### 4.7. Peptide Oxidation and Purification

The linear BmKDfsin3, His-BmKDfsin3, and ST2345 peptides were synthesized by GL Biochem Ltd. (Shanghai, China). The methods of oxidation and purification for BmKDfsin3 and His-BmKDfsin3 were as previously described by our group [46]. Briefly, BmKDfsin3 and His-BmKDfsin3 were oxidized by dissolving them with 0.2 M Tris-HCl (pH = 8.3) for 48 h at 25 °C in a shaker (50 rpm) at a concentration of 1 mg/mL. Reduced and oxidized peptides were analyzed and purified by HPLC on a C18 column (Elite HPLC, Dalian, China, 10 × 250 mm, 5 µm, 300 A). Typical setting of HPLC was a linear gradient from 5% to 95% CH_3_CN with 0.1% trifluoroacetic acid (TFA) in 60 min with a constant flow rate of 1–4 mL/min. The injection volume of HPLC was less than 5.0 mL every time. Targeted peptides were collected with detection at a wavelength of 230 nm and freeze-dried to save. Similarly, the HPLC condition of ST2345 peptide was the same as BmKDfsin3 and His-BmKDfsin3.

### 4.8. Statistics Analysis

Adobe Photoshop CS6 (Adobe Systems, Inc, San Jose, CA, USA) and Graphpad Prism 5 were used for statistical analysis. Data represented the mean ± SD of at least three independent experiments and *p* values calculated by the t-test of Student. Statistical significance were considered at a *p* value less than 0.05 (*, *p* < 0.05. **, *p* < 0.01. ***, *p* < 0.001).

## Abbreviations

CC_50_	50% cytotoxic concentration.
CHIKV	chikungunya virus.
DAAs	direct-acting antivirals.
DAPI	4′,6-diamidino-2-phenylindole.
DMSO	dimethyl sulfoxide.
EV71	enterovirus 71.
GAPDH	glyceraldehyde-phosphate dehydrogenase.
HBV	hepatitis B virus.
HCV	hepatitis C virus.
HPLC	high-performance liquid chromatography.
HSV	herpes simplex virus.
IC_50_	50% inhibitory concentration.
IL-1β	interleukin-1β.
LPS	lipopolysaccharide.
MAPK	mitogen-activated protein (MAP) kinase.
MKK	MAP kinase kinase.
MOI	multiplicity of infection.
MTT	3-[4,5-dimethylthiazol-2-yl]-2,5 diphenyl tetrazolium bromide.
NC	nitrocellulose.
PBS	phosphate buffered saline.
PKC	protein kinase C.
RNA	ribonucleicacid.
RSV	respiratory syncytial virus.
qPCR	quantitative PCR.
SD	standard deviation.
TNF-α	tumor necrosis factor-α.
